# Baseline metabolic signatures predict clinical outcomes in immunotherapy-treated melanoma patients: a pilot study

**DOI:** 10.3389/fimmu.2025.1536710

**Published:** 2025-08-01

**Authors:** Simona De Summa, Giuseppe De Palma, Veronica Ghini, Benedetta Apollonio, Ivana De Risi, Antonio Tufaro, Sabino Strippoli, Claudio Luchinat, Leonardo Tenori, Michele Guida

**Affiliations:** ^1^ Molecular Diagnostics and Pharmacogenetics Unit, IRCCS Istituto Tumori “Giovanni Paolo II”, Bari, Italy; ^2^ Institutional BioBank, Experimental Oncology and Biobank Management Unit, IRCCS Istituto Tumori “Giovanni Paolo II”, Bari, Italy; ^3^ Magnetic Resonance Center (CERM), University of Florence, Florence, Italy; ^4^ Department of Chemistry “Ugo Schiff”, University of Florence, Florence, Italy; ^5^ Rare Tumors and Melanoma Unit, IRCCS Istituto Tumori “Giovanni Paolo II”, Bari, Italy; ^6^ Giotto Biotech srl, Florence, Italy; ^7^ Consorzio Interuniversitario Risonanze Magnetiche di Metallo Proteine (CIRMMP), Florence, Italy

**Keywords:** immune checkpoint inhibitors, NMR, immunotherapy-treated melanoma patients, tumor-related metabolic fingerprints, serum metabolomic profiles, separate predictive risk score model

## Abstract

**Background:**

Immune checkpoint inhibitors (ICIs) have improved the metastatic melanoma (MM) treatment. However, a significant proportion of patients show resistance to immunotherapy, and predictive biomarkers for non-responders or high-risk recurring patients are currently lacking. Recent studies have shown that tumor-related metabolic fingerprints can be useful in predicting prognosis and response to therapy in various cancer types. Our study aimed to identify serum-derived metabolomic signatures that could predict clinical responses in MM patients treated with ICIs.

**Patients and methods:**

^1^H-NMR (proton nuclear magnetic resonance) was used to analyze the serum metabolomic profiles from 71 MM patients undergoing anti-PD-1 therapy (43 patients as first-line, 27 as second-line, 1 as third-line). Feature selection was applied to identify key metabolites within these profiles, to develop risk score models predicting overall survival (OS) and progression-free survival (PFS).

**Results:**

A multivariable model was used to identify distinct prognostic factors for OS. Negative factors included glucose, high-density lipoprotein (HDL) cholesterol, and apolipoprotein B-very low-density lipoprotein (ApoB-VLDL), whereas glutamine and free HDL cholesterol emerged as positive factors. They were then used to construct a risk score model able to stratify patients in prognostic groups. Similarly, a separate predictive risk score model for PFS was developed, focusing solely on glucose and apolipoprotein A1 (ApoA1) HDL. Threefold cross validation resulted in mean concordance indices of 0.72 and 0.74 for PFS and OS, respectively. Importantly, this analysis was replicated in patients who received first-line ICIs. Interestingly, the prognostic score for OS included glutamine, glucose, and LDL (low-density lipoprotein) triglycerides, whereas only glucose negatively influenced PFS. In this subset, the concordance indices increased to 0.81 and 0.9 for PFS and OS, respectively.

**Conclusions:**

Our data identified glycolipid signatures as robust predictors of distinct therapeutic outcomes in MM patients treated with ICIs. These results could pave the way for novel therapeutic approaches.

## 1 Introduction

Targeted therapies and immune checkpoint inhibitors (ICIs) have dramatically improved the treatment of metastatic melanoma (MM) in the past decade. Both approaches significantly prolong patients’ survival; however, targeted therapy can only be used in BRAF-mutated patients, which represent approximately 50% of the MM population. Moreover, most patients treated with BRAF/MEK inhibitors develop resistance to treatment due to the occurrence of MAPK pathway-activating mutations (1).

ICIs instead can be considered for all patients regardless of BRAF mutational status and have exhibited improved survival outcomes. Antibody-based inhibitors of programmed death protein 1 (PD-1) induce objective responses of approximately 40% in controlled clinical trials when used as a single agent, with only 10%–30% of the patients achieving long-term and sustained efficacy. As a result, approximately two-thirds of patients do not experience long-lasting clinical benefits due to developing primary or secondary resistance (2, 3).

Numerous ICI resistance mechanisms have been reported. Most of them involve the recruitment and activation of immunosuppressive cell populations in the tumor microenvironment (TME), and their interactions with stroma and extracellular matrix components (4).

In addition, accumulating evidence has highlighted the importance of cancer cell metabolic reprogramming and its capability to drive tumor growth and immunosuppression (5–7).

Interestingly, tumor-induced metabolic rewiring alters the metabolome (metabolites profile) of tissues and biological fluids, and several studies have shown that specific diseases are associated with different metabolomic fingerprints, suggesting their diagnostic and prognostic roles (8).

Metabolome is a dynamic entity that changes during tumor growth and progression (9, 10). Its analysis provides a snapshot of tumor-induced alterations, including the biomolecular mechanisms underlying response or resistance to specific treatments, and those related to the development of specific therapy-induced adverse effects (11, 12). Therefore, identifying specific metabolomic profiles holds promise for stratifying patients into distinct groups. This stratification could guide treatment decisions by predicting which patients are more likely to benefit from specific therapies, experience certain adverse events, or respond favorably to dietary interventions and/or combination therapies.

Nuclear magnetic resonance (NMR) spectroscopy represents a rapid (no sample preprocessing) and untargeted analytical approach to dissect metabolomic profiles (13). In this study, we used NMR to evaluate the metabolome from the serum of MM patients before anti-PD-1 treatment. We aimed to identify metabolic signatures predicting both disease-specific features and clinical outcomes of ICIs therapy.

## 2 Patients and methods

### 2.1 Patient population

We built an observational study cohort, by prospectively recruiting from January 2018 to September 2021, 71 MM patients treated with anti-PD-1 antibodies (nivolumab or pembrolizumab) according to the standard dose and schedule (14). In detail, pembrolizumab was used at 2 mg/kg or at a flat dose (200 mg every 3 weeks or 400 mg every 6 weeks, respectively), and nivolumab at 3 mg/kg every 2 weeks or at a flat dose (240 mg every 2 weeks or 400 mg every 4 weeks, respectively) until disease progression, unacceptable toxicity, or patient request.

The following data were collected for each patient: general features (age, sex, ECOG, body mass index (BMI), concomitant medications), disease characteristics (site of primary melanoma and metastases, basal LDH level, BRAF mutational state), treatment (any previous therapies), and clinical outcomes, including response to therapy, progression-free survival (PFS), and overall survival (OS) (**Table 1**). The study was approved by the local Ethics Committee of Istituto Tumori “Giovanni Paolo II” of Bari (prot. no 515/2015 CE) and conducted in accordance with the international standards of good clinical practice.

**Table 1:** Main patient characteristics and outcomes to checkpoint immunotherapy.

Patients features	Numbers
Median age at metastatic disease, years (range)	61 (31-92)
Sex, male/female, n (%)	39/32 (55/45)
ECOG performance status, median (range)	0 (1-2)
Body mass index	
Total population Responder Non-responder	26,83 25,1 27,06
Concomitant medication, n (%)	
Anti-diabetic drugs Hypocholesterolemic agents	7 (9,8%) 6 (8,4%)
Type of melanoma, n (%)	
Cutaneous Uveal Mucosal Unknown origin	57(80) 3 (4) 2 (3) 9 (13)
BRAF status, n (%)	
Mutated V600 Mutated not V600 Wild type	26 (37) 3 (4) 42(59)
LDH, n (%)	
<ULN >ULN Unspecified	39 (55) 29 (41) 3 (4)
Previous systemic therapy for metastatic disease, n (%)	
Yes No	28 (40) 43 (60)
Best response, n (%)	
ORR DCR	21 (30) 24 (34)
PFS median, months	3
OS median, months	8

ORR, overall response rate; complete response + partial response. DCR, disease control rate; complete response + partial response + stable disease > 6 months. PFS, progression-free survival; OS, overall survival.

### 2.2 Clinical assessment

The best objective response rate (BORR) was determined as the percentage of patients with an objective response (complete response [CR] or partial response [PR]) per RECIST guideline, version 1.1 (15). Stable diseases (SD) more than 6 months were evaluated together with CR and PR due to their similar survival rates. SD of less than 6 months was considered with progressive disease (PD). PFS was calculated as the time between the beginning of immunotherapy and tumor progression or last follow-up. OS was calculated as the length of time from the start of therapy and patient death or last follow-up.

### 2.3 Sample preparation

Serum was collected before ICI therapy by centrifugation of venous blood at 1,900g × 10 min within 30 min of collection. Blood withdrawal was conducted under fasting conditions exclusively in the morning between 8:00 and 9:00 am to avoid circadian variations. Before blood withdrawal, patients were not given drugs. No dietary indications had previously been given. Samples were immediately cryopreserved at −80°C at the Institutional Biobank of Istituto Tumori “Giovanni Paolo II”, Bari.

### 2.4 Metabolomic profiling using NMR analysis

Samples containing 1 ml of serum were sent at CERM-University in Florence and analyzed by ^1^H NMR-based metabolomics.

NMR samples were prepared and recorded according to standard procedures for serum/plasma samples for metabolomics analysis (16). NMR spectra were acquired using a Bruker 600 MHz spectrometer (Bruker Biospin) operating at 600.13 MHz of Larmor proton frequency and equipped with a PATXI ^1^H−^13^C−^15^N and ^2^H decoupling probe including a z-axis gradient coil, automatic tuning–matching (ATM), and an automatic, refrigerated sample changer (SampleJet, Bruker Biospin). A BTO 2000 thermocouple served to stabilize the temperature at approximately 0.1 K in the sample. Before measurement, samples were kept for 5 min in the NMR probe head, for temperature equilibration at 310 K.

For each sample, three one-dimensional ^1^H NMR spectra were acquired with water peak suppression and different pulse sequences: (*i*) NOESY 1Dpresat, which detects both the signals of small molecules—metabolites—and those of macromolecules—lipoproteins, proteins, and lipids; (*ii*) 1D CPMG, which selectively reveals the signals of metabolites; (*iii*) 1D diffusion-edited, which selectively reveals the signals of macromolecules. Free induction decays were multiplied by an exponential function equivalent to a 0.3-Hz line-broadening factor before applying Fourier transform. Transformed spectra were automatically corrected for phase and baseline distortions and calibrated at the glucose doublet at δ 5.24 ppm using TopSpin 4.1 (Bruker Biospin).

A total of 24 metabolites (**Supplementary Table S1**) were assigned in all the spectra and their concentrations analyzed. Metabolites were analyzed using the *In Vitro* Diagnostics research (IVDr) B.I. Quant-PS tool (Bruker, Biospin). The IVDr Lipoprotein Subclass Analysis B.I. LISA tool (Bruker, Biospin) was used to extract 114 parameters associated with lipoproteins (main parameters, calculated features, main fractions, subfractions, and particle numbers).

### 2.5 Data preprocessing and feature selection

Data were preprocessed using the *caret* R package. To perform feature selection, the Recursive Feature Elimination (RFE) method of the *caret* package has been applied to the normalized dataset, with 10-fold cross-validation. The RFE feature selection has been applied using BORR as a dependent variable. In detail, BORR was dichotomized grouping patients into “responder” and “not-responder”, including CR, SD, PR responses, and PD cases, respectively.

### 2.6 Statistical analysis

All statistical analyses were performed using R (version 4.2.1). Univariate and multivariate Cox-hazard regression models were fitted through R packages “survival” and “survminer”. R package “binda” was used to dichotomize variables.

#### 2.6.1 Risk score calculation

The significant features of the multivariate Cox hazard regression models and their coefficients were used to calculate risk score (RS), using the general formula:


RS=metabolite1 x β metabolite 1metabolite2 x β metabolite 2+metabolite3 x β metabolite 3

#### 2.6.2 Threefold cross validation

The risk scores underwent threefold cross-validation considering that sample size and concordance index (CI) were calculated through the “survcomp” R package.

#### 2.6.3 Survival curves

Patients were stratified according to the median values of RSs in “low risk” and “high risk” groups, and Kaplan–Meier curves were compared using the log-rank test. Graphs and forest plots were created using the “ggplot2” and “forestmodel” R packages respectively.

## 3 Results

### 3.1 Patient cohort

A total of 71 serum samples from MM patients treated with anti-PD-1 (nivolumab: 42 patients, pembrolizumab: 29 patients) were analyzed. There were 43 patients (61%) who received anti-PD-1 as first-line treatment. Of patients that received ICIs as second line, 22 (31%) were previously treated with BRAF and MEK inhibitors and 5 patients (7%) with ipilimumab. Only one patient (1%) received two previous lines of therapy (first line: target therapy, second line: ipilimumab). All the samples were collected before anti-PD-1 treatment.

Main patient characteristics included cutaneous melanoma 80%, LDH<ULN in 55% of patients, M1c stage in 31%, and BRAF V600 mutation in 37% of patients. Median age at metastatic disease was 61 years (range 31-92) and median ECOG: 0 (0-2) median BMI was 26.83 (**Table 1**). Only seven and six patients were given hypoglycemic and hypocholesterolemic medications, respectively, as concomitant treatments.

Of 71 patients assessed for response to anti PD-1, 10% (7 patients) had a complete response (CR), 20% (14 patients) had a partial response (PR), and 4% (3 patients) showed a long-term stable disease (SD), with 34% disease control rate (DCR) and 30% overall response rate (ORR). There were 45 patients (63%) who did not respond to immunotherapy, and 2 patients (3%) had an SD with a PFS of less than 6 months.

Median PFS was 3 and 4 months for the whole cohort and the ICI first-line-treated subgroup, respectively; median OS was 8 months for both subsets.

Four patients discontinued anti-PD-1 therapy due to treatment-related toxicity, whereas five patients interrupted therapy after achieving a confirmed complete response and four due to a very good partial (near-complete) response and completing at least 2 years of treatment, in line with clinical practice.

To characterize the metabolomic profiles of MM patients, baseline serum samples were subjected to untargeted metabolomic analysis using ¹H-NMR spectroscopy. This approach enabled the quantification of a wide range of low-molecular-weight metabolites, and lipoprotein-related parameters (**Supplementary Table S1**). The resulting quantitative data, encompassing a metabolic fingerprint of each sample, was employed for subsequent bioinformatic analyses.

### 3.2 Metabolomic-based risk models for melanoma patients treated with ICIs

Using the RFE method for feature selection, we identified the top 20 metabolites most effective in discriminating between responder and non-responder patients (**Supplementary Figure 1**). These top 20 features, encompassing both lipoproteins and metabolites, were then dichotomized based on their median values and analyzed using a univariate Cox regression model. Our results showed that glucose (HR: 2.8, 95% CI: 1.52-5.15) and ApoB VLDL (HR: 2.75, 95% CI: 1.51-5.02) were associated with greater risk of death, whereas glutamine (HR: 0.34, 95% CI: 0.18-0.62), ApoA1 HDL (HR: 0.44, 95% CI: 0.24-0.81), HDL cholesterol (HR: 0.5, 95% CI: 0.27-0.91), free HDL cholesterol (HR: 0.48, 95% CI: 0.27-0.88), and ApoA1 (HR: 0.51, 95%CI: 0.28-0.93) were associated with a lower risk (**Table 2**).

**Table 2:** Univariate Cox hazard regression model for OS of the entire cohort.

Characteristic	N	HR^1^	95% CI^1^	p-value
Basal LDH*	68			
1		—	—	
2		1.74	0.96, 3.13	0.066
Glucose_d	71			
0		—	—	
1		2.80	1.52, 5.15	<0.001
Glutamine_d	71			
0		—	—	
1		0.34	0.18, 0.62	<0.001
HDL.Chol_d	71			
0		—	—	
1		0.50	0.27, 0.91	0.023
Apo.A1.HDL_d	71			
0		—	—	
1		0.44	0.24, 0.81	0.008
Apo.A1_d	71			
0		—	—	
1		0.51	0.28, 0.93	0.028
FreeChol.HDL.1_d	71			
0		—	—	
1		0.48	0.27, 0.88	0.018
Apo.B.VLDL_d	71			
0		—	—	
1		2.75	1.51, 5.02	<0.001

^1^HR, hazard ratio; CI, confidence interval.

We also evaluated baseline LDH values from medical records, dichotomized above and below the normal range. We found a statistical trend with worse survival for patients with higher LDH levels (HR: 1.74, 95% CI: 0.96-3.13).

**Figure 1:**
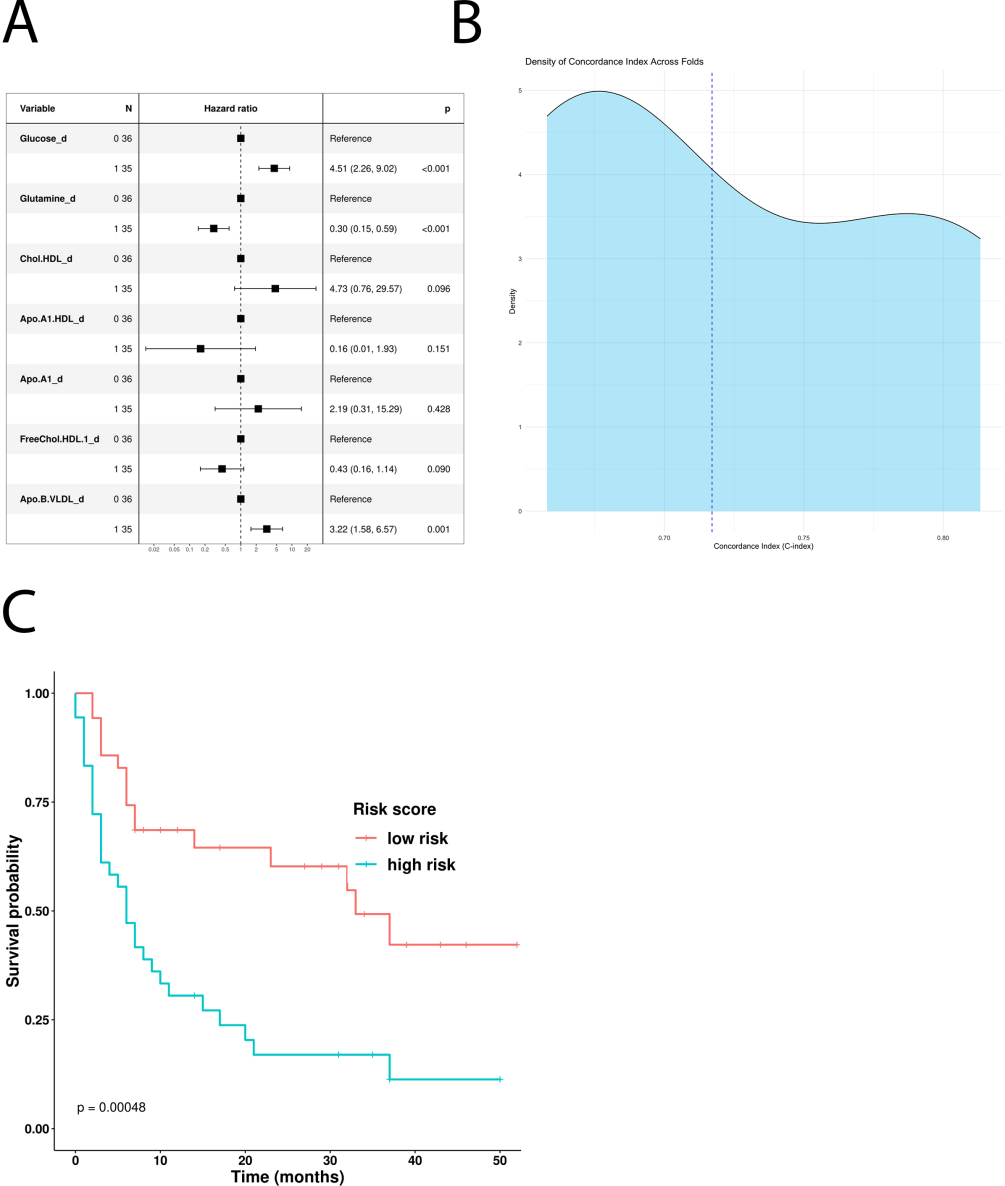
Impact of the univariate significant features on OS. **(A)** Multivariate Cox-hazard regression model; **(B)** threefold cross validation of OS RiskScore depicting density of the concordance index; **(C)** Kaplan–Meier survival curves stratifying patients according to the risk score calculated using the multivariate model.


A multivariate COX-hazard regression model revealed that glucose, glutamine, HLD.chol, FreeChol.HDL, and APO.B.VLDL are independent prognostic factors (**Figure 1A**), and so they were used to calculate a prognostic risk score.

$$RiskScoreOS\;=\;\left(1.68\;*\;Glucose\right)\;+\;\left(-1.34\;*\;Glutamine\right)\;+\;\left(1.93\;*\;HDL.Chol\right)\;+\;\left(-1.24\;*\;FreeChol.HDL\right)\;+\;\left(1.3\;*\;APO.B.VLDL\right)$$

The model has been confirmed also excluding patients which underwent hypoglycemic/hypocholesteromic agents (**Supplementary Figure 3**).

The model underwent threefold cross validation which evidenced a mean CI of 0.74, as displayed in **Figure 1B**. Comparison of baseline clinical features stratifying according to RiskScoreOS evidenced that the distribution of the number of metastatic sites differs significantly between the low-risk and high-risk groups (p = 0.044). In detail, among patients with fewer than three metastatic sites, a higher proportion belongs to the high-risk group (64%) compared with the low-risk group (40%). Conversely, among those with three or more metastatic sites, 60% are in the low-risk group versus 36% in the high-risk group (**Supplementary Table 3**).

Following stratification by the median risk score, Kaplan–Meier curves were used to visualize the OS of patients in the high- and low-risk groups. Interestingly, the “low risk” group showed a statistically significant survival advantage compared with the “high risk” group (p-value: 0.00048, **Figure 1C**).

**Figure 2:**
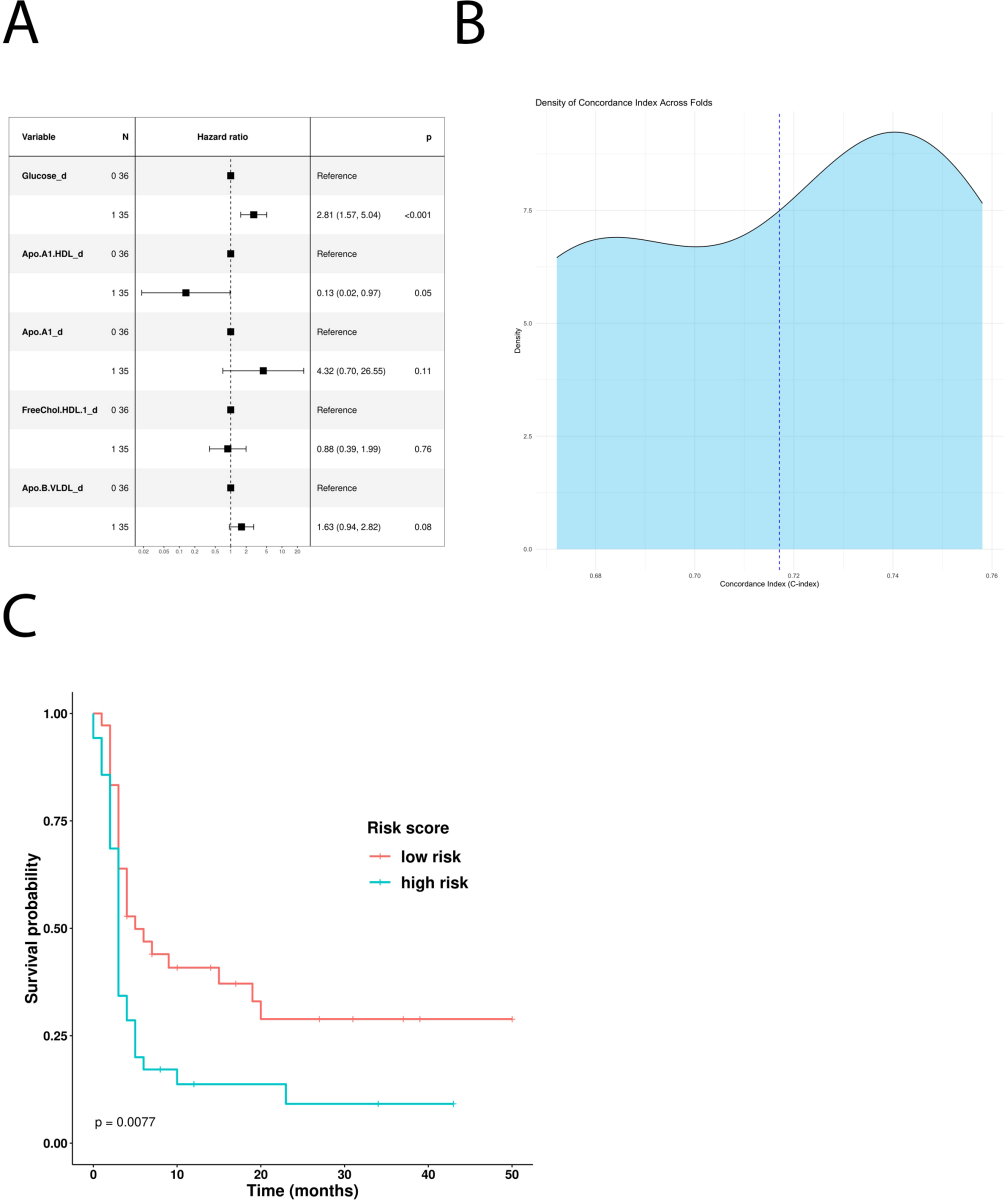
Impact of the univariate significant features on PFS. **(A)** Multivariate Cox-hazard regression model; **(B)** threefold cross validation of PFS RiskScore depicting density of the concordance index; **(C)** Kaplan–Meier survival curves stratifying patients according to the risk score calculated through the multivariate model.

To understand the impact of baseline serum metabolome on PFS, we used a univariate Cox hazard regression model showing that glucose, ApoA1, HDL cholesterol, HDL ApoA1, free HDL cholesterol, and LDL triglycerides were significantly associated with disease progression (**Table 3**). According to these results, the multivariate model identified glucose and ApoA1 as negative risk factors, whereas HDL ApoA1 showed a protective role (**Figure 2A**). The model has been confirmed also excluding patients which underwent hypoglycemic/hypocholesteromic agents (**Supplementary Figure 4**).

**Table 3:** Univariate Cox hazard regression model for PFS of the entire cohort.

Characteristic	N	HR^1^	95% CI^1^	p-value
Glucose_d	71			
0		—	—	
1		2.68	1.54, 4.67	<0.001
Chol.HDL_d	71			
0		—	—	
1		0.59	0.35, 1.02	0.057
Apo.A1.HDL_d	71			
0		—	—	
1		0.48	0.28, 0.83	0.008
Apo.A1_d	71			
0		—	—	
1		0.58	0.34, 1.00	0.048
FreeChol.HDL.1_d	71			
0		—	—	
1		0.57	0.33, 0.98	0.041

^1^HR, hazard ratio; CI, confidence interval.

The use of these variables to build a new PFS Risk model (Risk score PFS = (1.03*Glucose)+(-2.01*Apo.A1.HDL)) highlighted that high-risk patients have shorter PFS compared with the low-risk ones (p-value: 0.0077) (**Figure 2C**). PFS Risk Score reached a mean CI of 0.72 (**Figure 2B**). Comparison of baseline clinical features stratifying according to Risk score PFS did not evidence no statistically significant association (**Supplementary Table 4**).

### 3.3 Metabolomic-based risk models for melanoma patients treated with first-line ICIs

To better understand how the baseline serum metabolome impacts response to immunotherapy, and to identify potential predictive markers, we built a metabolomic-based risk model for the restricted group of MM patients who received first-line ICI treatment (n=43). The top 20 features were selected using the same RFE approach described earlier (**Supplementary Figure 2**).

**Figure 3:**
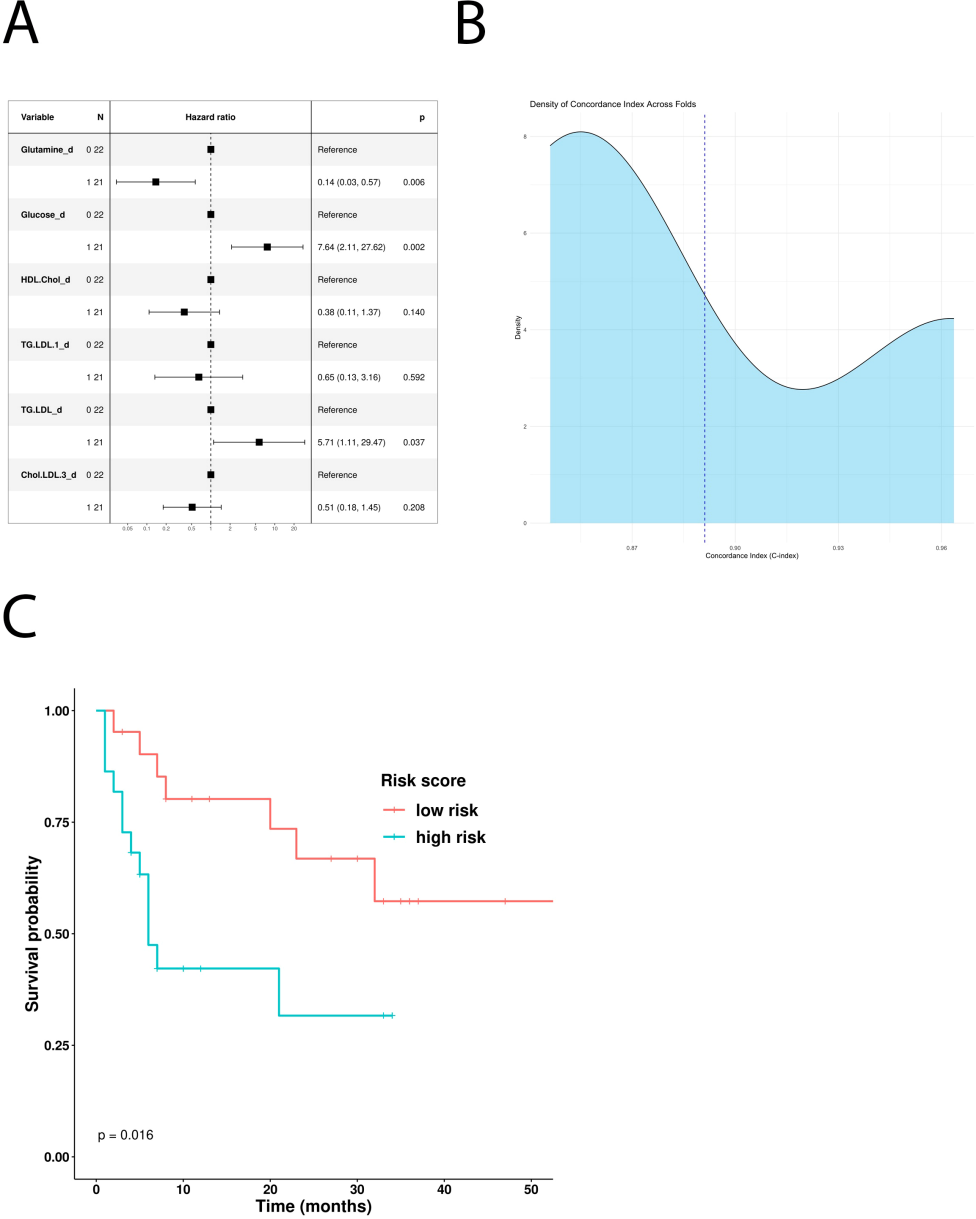
Prognostic role of the different metabolites in patients treated with first-line ICI. **(A)** Multivariable Cox-hazard regression model; **(B)** threefold cross validation of OS RiskScore depicting density of the concordance index; **(C)** Kaplan–Meier survival curves comparing OS of patients stratified according to the risk score.

Glucose, glutamine, HDL cholesterol, LDL triglycerides, and free LDL cholesterol were all identified as significant features in the OS-based univariate COX hazard regression model (**Table 4**). Multivariate analysis highlighted glucose and LDL triglycerides as negative prognostic factors and glutamine as positive one (**Figure 3A**). Kaplan–Meier curves comparing low- and high-risk subgroups (ICI-Risk ScoreOS = (2.03*Glucose)+(-1.97*Glutamine) + (1.74*TG.LDL)) confirmed that the prognostic model was able to significantly stratify patients (**Figure 3C**), with a mean CI after threefold cross validation of 0.9 (**Figure 3B**).

**Table 4:** Univariate Cox hazard regression model for OS of first-line ICI-treated MM patients.

Characteristic	N	HR^1^	95% CI^1^	p-value
Glutamine_d	43			
0		—	—	
1		0.24	0.09, 0.64	0.004
Glucose_d	43			
0		—	—	
1		5.27	1.90, 14.6	0.001
HDL.Chol_d	43			
0		—	—	
1		0.31	0.12, 0.80	0.016
TG.LDL_d	43			
0		—	—	
1		3.69	1.41, 9.70	0.008
Chol.LDL.3_d	43			
0		—	—	
1		0.36	0.14, 0.92	0.033

^1^HR, hazard ratio; CI, confidence interval.

**Figure 4:**
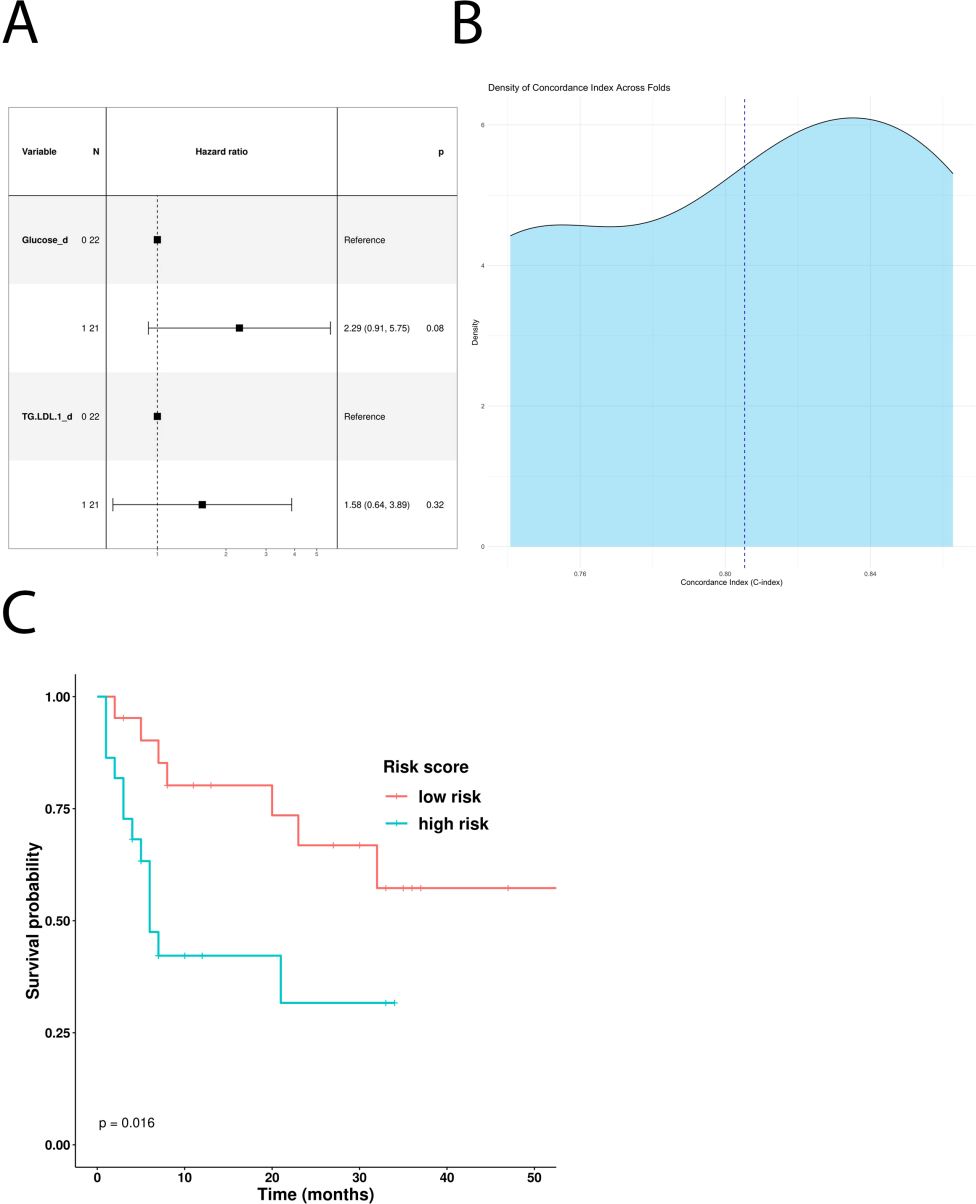
Predictive role of the different metabolites in patients treated with first-line ICI. **(A)** Multivariable Cox-hazard regression model; **(B)** threefold cross validation of PFS RiskScore depicting density of Concordance index; **(C)** Kaplan–Meier survival curves comparing PFS of patients stratified according to the risk score.

In addition, the univariate Cox hazard regression model revealed glucose and LDL triglycerides as independent negative predictive factors for PFS (**Table 5**). However, multivariate COX analysis showed only glucose as an independent prognostic factor (**Figure 4A**). According to this result, MM patients treated with first-line immunotherapy and stratified using a glucose-based risk score (ICI-Risk ScorePFS = (0.82*Glucose)), with a mean CI of 0.81 (**Figure 4B**), showed statistically significant differences in Kaplan–Meier curves (**Figure 4C**).

**Table 5:** Univariate Cox hazard regression model for PFS of first-line ICI-treated MM patients.

Characteristic	N	HR^1^	95% CI^1^	p-value
Glucose_d	43			
0		—	—	
1		3.03	1.45, 6.32	0.003
TG.LDL.1_d	43			
0		—	—	
1		2.59	1.26, 5.33	0.010

^1^HR, hazard ratio; CI, confidence interval.

## 4 Discussion

ICI immunotherapy has significantly broadened the treatment landscape for MM, with a significant proportion of patients obtaining long-lasting and deep responses (2, 3). However, despite extensive research, validated clinical-grade biomarkers capable of discriminating responsive or resistant cases to ICI have not been identified yet (4, 17–19). Moreover, additional studies are required to identify resistance mechanisms and to develop novel combination treatments improving currently available therapies.

Together with tumor cell-intrinsic features (e.g., loss of antigen presentation or expression of immunosuppressive molecules), antitumor immune responses are also impaired by several other mechanisms mediated by the tumor microenvironment (TME), including stromal barriers, recruitment of immunoregulatory cells, hypoxia, and metabolic imbalance (20).

Metabolic alterations have been increasingly recognized as a crucial hallmark of cancer, regulating tumorigenesis, proliferation, survival, invasiveness, and immunosuppressive capabilities (21). Indeed, cancer cells are characterized by uncontrolled and non-finalistic proliferation, which requires large amounts of nutrients and energy and triggers continuous changes in their metabolic profiles. Metabolic reprogramming is essential to supporting tumor growth even in adverse conditions, such as limited oxygen and nutrient availability. Dysregulation in carbohydrate, amino acid, and lipid metabolism are distinctive features of cancer cells (22). Metabolic rewiring alters metabolite concentrations in the TME, affecting the activation status and recruitment of different immune cells, thus potentially interfering with clinically relevant responses to immunotherapy (6).

Serum metabolomic signatures can be easily quantified, and they could provide an indirect snapshot of the TME metabolic status. Our study aimed to develop a metabolomic-based model capable of predicting clinical outcomes in MM patients treated with ICIs.

We profiled the serum of 71 MM patients at baseline (before ICI treatment) using NMR spectroscopy, an analytical approach offering minimal sample preparation and high experimental reproducibility, thus potentially suitable for clinical applications (23). NMR analysis has been extensively used to detect the metabolic behavior of melanoma cell lines and xenograft models (24, 25).

In our study, we applied for the first time NMR-based metabolomics on the serum of MM patients treated with ICIs to build risk score models predicting OS and PFS. A similar approach has been previously applied in patients with non-small-cell lung cancer (26). Our data demonstrated that glucose is a high-risk factor. In line with our findings, a consistent body of evidence indicates that elevated blood glucose levels in MM, akin to those observed in diabetes, exert a negative prognostic impact and detrimentally influence the efficacy of checkpoint inhibitor therapy (27–29). Notably, in a large cohort study of MM patients treated with immune checkpoint inhibitors, Jan et al. (30) demonstrated that type 2 diabetes was associated with significantly worse survival outcomes, with the most pronounced detrimental effect observed in patients with a low body mass index.

An elevated serum glucose level could be indicative of high glucose availability in the TME, which fuels cancer cell proliferation through the Warburg effect (31) and immunosuppression (6). Increased cancer cell metabolism reduces glucose availability for tumor-infiltrating CD8 T cells, inducing their exhaustion (32). More importantly, by increasing their glycolytic activity, tumors release large amounts of lactate in the extracellular space, which inhibits T and NK cell proliferation and cytotoxicity, while promoting Treg and myeloid-derived suppressor cell (MDSC) survival (33, 34). A key enzyme in lactate metabolism is lactate dehydrogenase (LDH). As expected, elevated LDH serum levels at baseline are predictive of shorter survival in patients with melanoma also in our cohort of patients. LDH is a complex biomarker associated with the activation of several oncogenic signaling pathways, as well as with increased metabolic activity, invasiveness, and reduced immunogenicity (35). Our data suggest that high glucose consumption and increased LDH activity foster immunosuppression and reduced ICI efficacy in MM patients, as recently observed in an anti-PD-1 + anti-LAG-3 retrospective study (27). In another study by Triozzi and colleagues (36), the baseline metabolomic profile was assessed in 40 patients with MM undergoing treatment with PD-1 inhibitors. The identified metabolomic signatures were correlated with the oxidative and glycolytic activity of circulating T lymphocytes, as well as with the expression of genes associated with metabolic functions. In line with our data, Triozzi and colleagues demonstrated that lower pretreatment glycemic values, increased oxidative metabolism in circulating immune cells, and higher expression of Glut-14, an intracellular glucose transporter, were predominant in anti-PD-1 responder patients.

Our data have also highlighted the protective role of glutamine. Metabolomic studies performed in melanoma patient-derived xenografts have revealed an intriguing inverse relationship between glutamine levels and histone methylation within cancer cells (36). This epigenetic interplay, influenced by the depletion of α-ketoglutarate, has been implicated in both melanoma differentiation and resistance to therapy under conditions of low glutamine levels (37). Conversely, a direct association between glutamine metabolism and autophagy has been identified, suggesting a potential enhancement of antigen exposure that could synergize with ICI (38, 39). Furthermore, high levels of glutamine and an increase of its metabolism could boost cytotoxic and pro-inflammatory capabilities of T lymphocytes (40).

The risk model we developed also contains specific lipid metabolites. Together with glucose and glutamine, ApoA-I holds a key position in the lipidic metabolic network. Embedded in various biological contexts, ApoA-I is the primary protein constituent of plasma HDL and it has been linked to survival across diverse human cancers. Indeed, it affects clinical outcomes by shaping the tumor microenvironment and by influencing immune system antitumor activity. *In vivo* studies have demonstrated the potent anti-tumorigenic effects of ApoA1, including significant suppression of tumor growth and metastasis in mouse tumor models (41, 42). Additionally, pretreatment levels of ApoA1 are predicting favorable outcomes in patients undergoing anti-PD1 therapy for metastatic colorectal cancer, intrahepatic cholangiocarcinoma, and nasopharyngeal carcinoma (43–45). On the opposite side of high-density lipoproteins are low-density lipoproteins, which have been associated with resistance to immune checkpoint inhibitors by suppressing T lymphocytes and driving tumor anti-apoptotic mechanisms mediated by heme oxygenase-1 (46). In our risk model, ApoB-VLDL has a negative predictive role, which has already been reported to be involved in the regulation of the expression of multiple genes in the development of hepatocellular carcinoma (47).

Our study fits within the comprehensive analysis of the influence of lipid metabolism on tumor immune response. Within this framework, the double-edged sword role of various lipid components in tumor immunity becomes apparent (48–50). In both melanoma and other potentially responsive tumors to ICIs, a contradictory influence is exhibited by pre-therapy levels and modifications of cholesterol, triglycerides, and oleic and palmitic acids (51, 52).

In this regard, rather than focusing solely on individual lipid components, our experience suggests that a risk formula capable of weighing the influence of individual metabolites within the general metabolomic context allows for a more faithful representation of each component’s role. This distinction has been necessary in the absence of a statistically significant difference between the BMI of responsive and non-responsive patients, thus lacking a clear reflection of obesity on these metabolic parameters.

It is important to recognize that the metabolic profile underlying this risk model, characterized primarily by hyperglycemia and hyperlipidemia, has been shown to be associated with poorer outcomes in patients treated with PD-1 inhibitors, regardless of tumor burden classification. Using the “three metastatic sites” threshold, which is based on prior meta-analyses of prospective clinical trials in MM (53, 54), we observed that fewer than three metastatic sites correlated with worse outcomes in our cohort. While this finding may seem counterintuitive at first glance, it is crucial to emphasize that prognosis and response to immunotherapy are often more strongly influenced by the site and extent of metastases, rather than their sheer number (55). This is particularly relevant in the context of our retrospective analysis, which is based on a real-world patient cohort that includes a significant proportion (22 patients) with M1d stage disease.

While this observation lends further support to the biological rationale behind our findings, it is essential to acknowledge that hyperglycemia and hyperlipidemia have also been associated with reduced OS, independent of tumor burden, as previously demonstrated in non-oncologic cohorts (56, 57). This represents a potential confounding factor in our analysis. However, the consistency of our data, evidenced by the correlation between glucose levels and both OS and PFS, as well as the confirmation of similar findings in existing literature, reinforces the robustness of our model.

The metabolomic fingerprints identified by our NMR analysis suggest that a set of pharmacological and dietary interventions could synergize with ICI and overcome therapeutic resistance. In this scenario, a repurposing strategy has been recently launched for hypoglycemic drugs as metformin (58), hypercholesteremic, and other lipid-lowering drugs such as statins and PCSK9 inhibitors (59, 60) and beta blockers (61), all of which leverage metabolism to steer the course of therapeutic response in favorable direction.

Several limitations are worth noting in our analysis. First, the sample cohort was relatively small, with only 71 patients characterized by treatment heterogeneity (43 of the 71 patients received ICI as first-line therapy). Second, our unselected real-world population exhibited lower ORR, PFS, and OS compared with published clinical trials. This could be attributed to factors such as prior lines of therapy and a higher frequency of negative prognostic factors, like elevated LDH levels (observed in 41% of patients). Additionally, while circulating metabolite changes remain a valuable biomarker, they may reflect systemic alterations arising from cross-organ communication rather than being exclusively tumor-derived. This systemic interplay can complicate the attribution of specific metabolic signatures directly to tumor activity, highlighting the need for integrative approaches to dissect tumor-specific versus host-related metabolic responses. Finally, our analysis is limited to a single baseline quantification of the serum metabolome and it does not explore potential changes in metabolic signatures over time during ICI treatment. Therefore, caution should be exercised when interpreting the relationship between metabolic parameters and clinical outcomes in MM patients treated with ICIs. Nonetheless, the promising results of the threefold cross-validation, with a high concordance index, particularly in the subset of first-line ICI-treated patients, suggest the need for a well-designed clinical trial to further validate and define the application of prognostic and predictive risk scores.

## 5 Conclusions

Predictive biomarkers for immunotherapy are related to a mosaic of different factors. Indeed, a complex combination of clinical features, serum factors, tumor and immune cell heterogeneity, genetic signatures, and other TME-related elements could all help to define an immunotherapy-responsive or resistant patient. Along this line, our data indicate that also baseline serum-derived metabolic fingerprints can be used to calculate metabolic risk scores with a prognostic value in MM patients treated with ICI. Further research is needed to verify whether these data can be an easily applicable tool in clinical practice.

## Data Availability

The original contributions presented in the study are included in the article/**Supplementary Material**. Further inquiries can be directed to the corresponding author.
