# Supplementary material for: Baseline metabolic signatures predict clinical outcomes in immunotherapy-treated melanoma patients: a pilot study
Source: Front Immunol. 2025 Aug 1;16:1536710. doi: 10.3389/fimmu.2025.1536710 (PMC12354368; doi:10.3389/fimmu.2025.1536710)
Supplement: Supplementary file 1 [file Table1.docx]

**Supplemental data**

**Table S1.** List of metabolites and lipoprotein-related parameters quantified in serum samples.

**Table S2**. Comparison of clinical parameters according to RiskScoreOS stratification.

**Table S3. Comparison of clinical parameters according to Risk score PFS stratification.**

**Figure S1.** Expression of the top 20 identified metabolites most effective in discriminating between responder (green) and non-responder (red) patients across the entire cohort of 71 MM patients.

**Figure S2.** Expression of the top 20 identified metabolites most effective in discriminating between responder (green) and non-responder (red) patients across the resticted cohort of 43 MM patients treated with first-line ICIs.

**Figure S3. OS Multivariate Cox hazard regression analysis removing patients following hypoglycemic and hypocholesterolemic medications**

**Figure S4. PFS Multivariate Cox hazard regression analysis removing patients following hypoglycemic and hypocholesterolemic medications**

**Supplementary Figure 1**


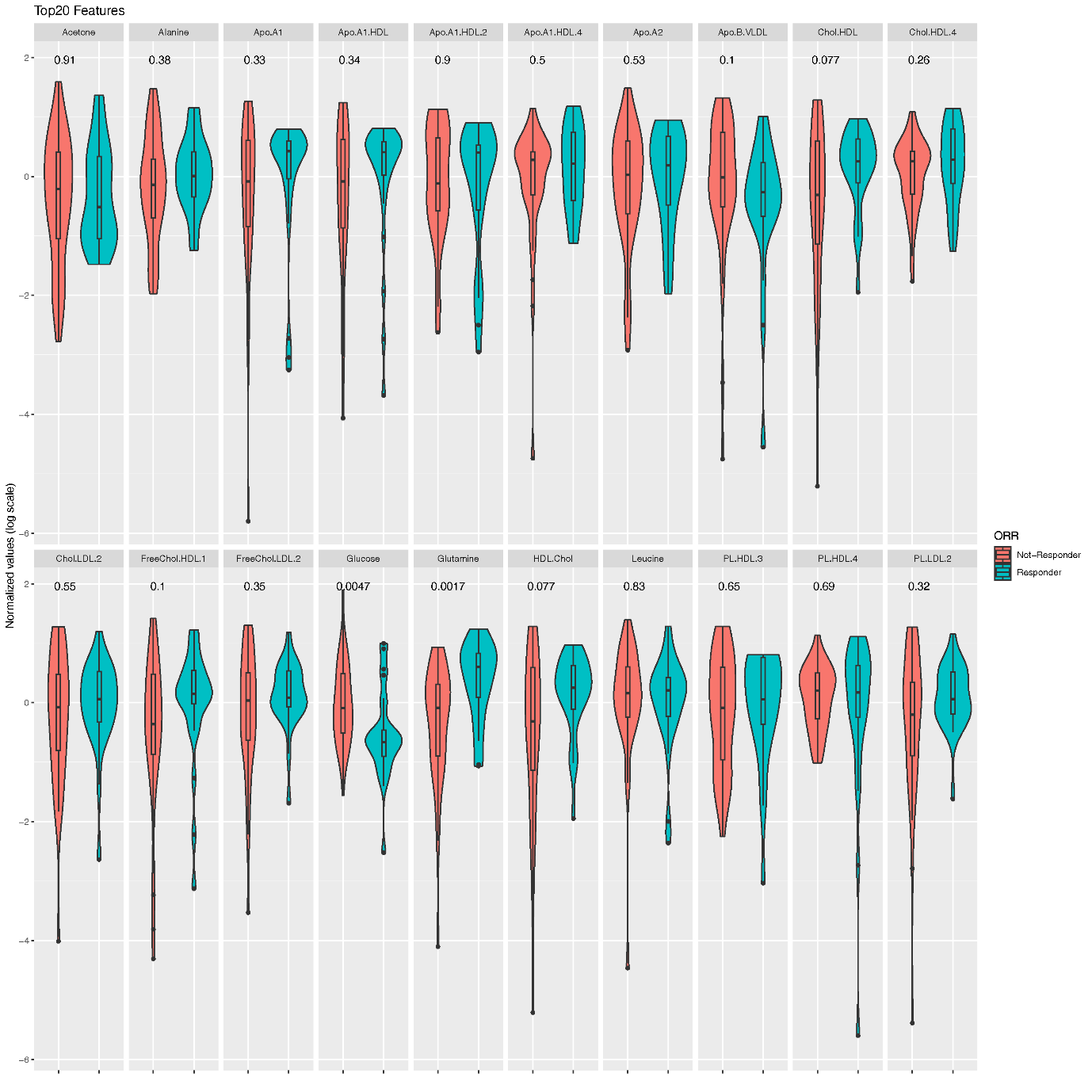


**Supplementary Figure 2**


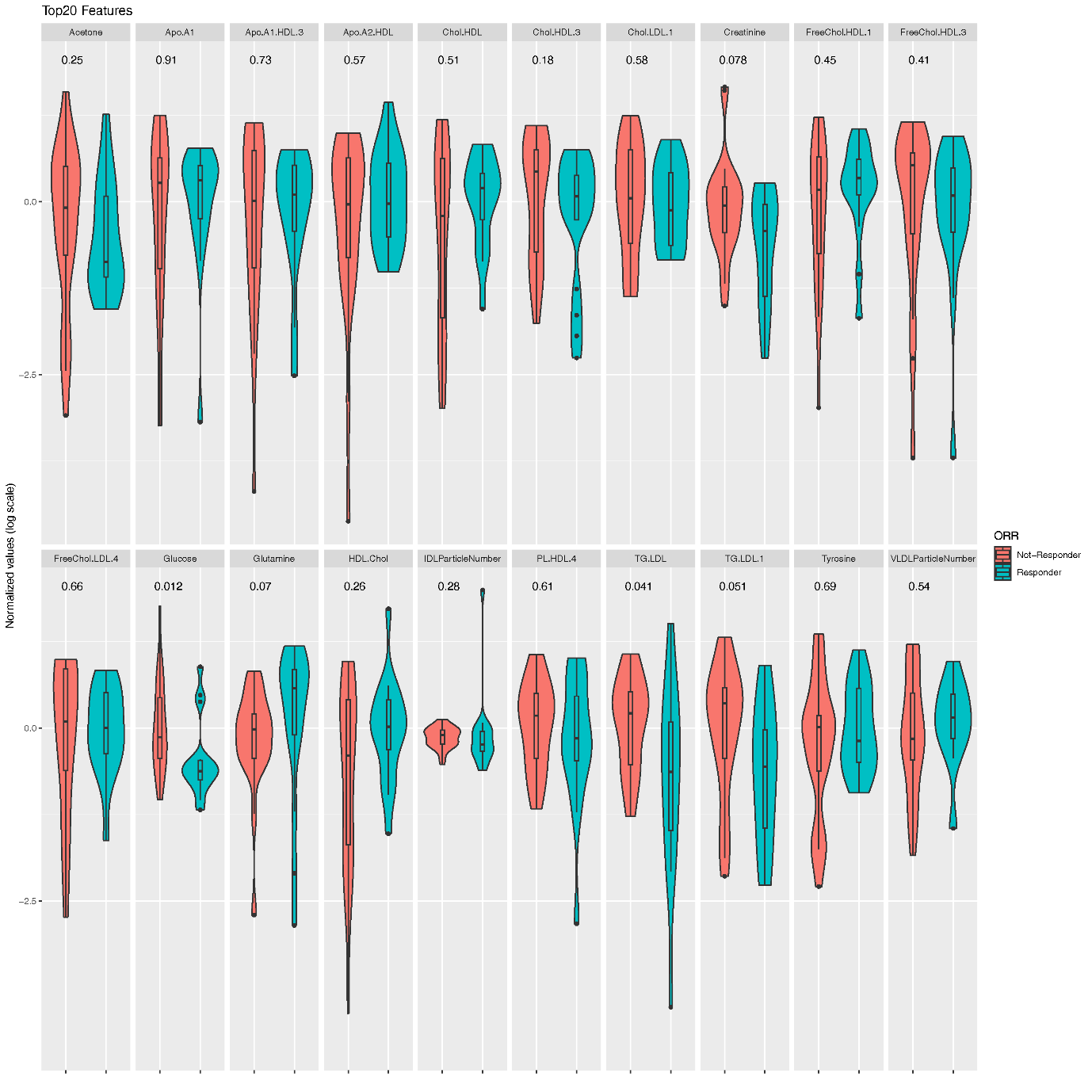


**Supplementary Figure 3**

**Supplementary Figure 4**

| **Characteristic** | **Overall**  N = 71^1^ | **Low Risk**  N = 35^1^ | **High Risk**  N = 36^1^ | **p-value**^2^ |
| --- | --- | --- | --- | --- |
| **BMI** | 32 (16, 48) | 31 (16, 45) | 37 (15, 49) | 0.6 |
| **Liver.metastatis** |  |  |  | 0.2 |
| No | 56 (79%) | 30 (86%) | 26 (72%) |  |
| Yes | 15 (21%) | 5 (14%) | 10 (28%) |  |
| **Nr.of.metastatic.sites** |  |  |  | 0.044 |
| <3 | 37 (52%) | 14 (40%) | 23 (64%) |  |
| >=3 | 34 (48%) | 21 (60%) | 13 (36%) |  |
| **BRAF.status** |  |  |  | >0.9 |
| mut | 29 (41%) | 14 (41%) | 15 (42%) |  |
| wt | 41 (59%) | 20 (59%) | 21 (58%) |  |
| **NRAS.status** |  |  |  | 0.3 |
| mut | 38 (54%) | 21 (60%) | 17 (47%) |  |
| wt | 33 (46%) | 14 (40%) | 19 (53%) |  |
| **Anti-PD1 resistance** |  |  |  | 0.3 |
| None | 19 (26.8%) | 10 (52.6%) | 9 (47.4%) |  |
| Primary | 39 (54.9%) | 21 (53.8%) | 18 (46.2%) |  |
| Secundary | 13 (18.3%) | 4 (30.8%) | 9 (69.2%) |  |
| ^1^Median (Q1, Q3); n (%) | | | | |
| ^2^Wilcoxon rank sum test; Fisher's exact test; Pearson's Chi-squared test | | | | |
|  | | | | |

**Supplementary Table 2**

**Supplementary Table 3.**

| **Characteristic** | **Overall**  N = 71^1^ | **Low Risk**  N = 36^1^ | **High Risk**  N = 35^1^ | **p-value**^2^ |
| --- | --- | --- | --- | --- |
| **BMI** | 26.8 (23.9, 29.4) | 26.3 (23.3, 29.2) | 26.9 (24.6, 31.1) | 0.3 |
| **Liver.metastatis** |  |  |  | 0.13 |
| No | 56 (79%) | 31 (86%) | 25 (71%) |  |
| Yes | 15 (21%) | 5 (14%) | 10 (29%) |  |
| **Nr.of.metastatic.sites** |  |  |  | 0.7 |
| <3 | 37 (52%) | 18 (50%) | 19 (54%) |  |
| >=3 | 34 (48%) | 18 (50%) | 16 (46%) |  |
| **BRAF.status** |  |  |  | 0.8 |
| mut | 29 (41%) | 14 (40%) | 15 (43%) |  |
| wt | 41 (59%) | 21 (60%) | 20 (57%) |  |
| **NRAS.status** |  |  |  | 0.5 |
| mut | 38 (54%) | 18 (50%) | 20 (57%) |  |
| wt | 33 (46%) | 18 (50%) | 15 (43%) |  |
| **Anti-PD1 resistance** |  |  |  | 0.9 |
| None | 19 (26.8%) | 9 (47.3%) | 10 (42.7%) |  |
| Primary | 39 (54.9%) | 20 (51.3%) | 19 (48.7%) |  |
| Secundary | 13 (18.3%) | 7 (53.9%) | 6 (46.1%) |  |
| ^1^Median (Q1, Q3); n (%) | | | | |
| ^2^Wilcoxon rank sum test; Fisher's exact test; Pearson's Chi-squared test | | | | |
